# Prof. Brainard’s Prize Essay

**Published:** 1855-03

**Authors:** 


### EDITORIAL.

*Prof. Brainard's Prize Essay.*

Under the head of critical analysis, the New York Journal of Medicine and Collateral Sciences, for January 1855, contains a notice of the last volume of transactions of the American Medical Association, several pages of which are devotod to Prof. Brainard's Prize Essay on the treatment of ununited fractures &c. With the comments of the writer, on the merits of the Essay we have nothing to do; but he introduces the subject with the following unjust, and in some respects erroneous remarka, viz:

"This pamphlet, of which our Prize Essay is but a translation was originally presented to the Society of Surgery, of Paris, by the author, and on the strength of which, we believe, he was elected a corresponding member of the society. In the early part of the year 1854, it was published in Paris, under the imprint of J. B. Bailiere, in its present form, and, as it would appear, previously to its being submitted to the American Medical Association.

The precedent established by this procedure, if followed out in future, resolves the prize committee of the Associations, into a committee of judges on recent publications—thus preventing the important object contemplated in the appointment of the committee. This double triumph of his Essay must be peculiarly gratifying to the author, as evincing a wide appreciation of his talents and investigations. Yet we can but feel that, in submitting it under the injunction of secresy after its foreign circulation, to the American Medical Association for its annual prize, he has not had sufficient regard for its national character and reputation. Representing in its Annual Transactions the progress of American Medicine, it but illy subserves this high purpose, when its prizes are awarded to essays already deferentially submitted to the criticisms of foreign societies, and which take the honor, if honor there be, in first publishing them to the world."

The plain inferences to be drawn from the foregoing paragraph are, that the same Essay which was presented by Prof. Brainard to the Prize Committee of the American Medical Association, was first submitted by him to the Society of Surgery of Paris; that such essay served as the basis of his election as corresponding member of said society; that said society had the honor, "if honor there was," of first publishing the Essay to the *world*; and that its subsequent presentation to the Prize Committee of the American Medical Association, was made without "sufficient regard for its national character and reputation"—in other words, it was an imposition on the Association. Now it so happens that not one of these inferences or allegations are correct.

The paper published by Prof. Brainard in Paris under the title of "Memoire sur le Traitement des Fractures non Reunies et des Difformites des Os," was not the *same identical* paper submitted to the American Medical Association. The one is not simply a literal translation of the other. Neither did that paper furnish the basis of his election as corresponding member; nor was it ever published by that society. The simple facts in relation to the whole matter are as follows: Prof. Brainard had been for some time pursuing certain experimental investigations, including the effects of injecting certain substances into the blood, tissues and cavities of the body; the efficacy of different substances in neutralizing the poison of serpents; and the effects of wounds and the presence of foreign bodies, on the growth of bone and the formation of callous. To pursue further these and other investigations, he visited Europe in the Autumn of 1853, and spent most of the following winter in Paris. Soon after his arrival he presented to the Society of Surgery, a paper on Injections of Iodine into the cavities and tissues of the body for the cure of spina bifida, Hydrocephalus, œdema and erysipelas, and at the same time became a candidate for election as corresponding member.

In the mean time he continued his researches in relation to diseased and deformed bones, and published them under the title above given, causing to be printed only a very limited number of copies for the purpose of distributing to personal friends. Having by this very limited publication placed his views on record at a proper date, Prof. B. reproduced them in a more carefully elaborated form, in our own language and presented them to the prize committee of the American Medical Association. Such are the facts in the case. But even if the Essay presented to the association had been an exact copy of the paper published a few months before in Paris, it would have been neither improper nor without precedent.

Thus the Essay of Dr. H. Gray, "on the structure and use of the spleen," which received the award of a Medal from the London Committee, had been regularly published one or two years previous; and yet the rules of the committee making the award in relation to *secrecy* were the same as those prescribed by our Association. And we have the very best authority for saying that there is scarcely a society of note in Paris or elsewhere on the Continent of Europe which has not often done the same thing. We cannot help thinking that the New-York reviewer has but partially apprehended the true object of establishing a Prize Committee in connection with our National Association. If said committee is to receive only papers made up exclusively of *new matter*, no part of which has ever been made public, it is easy to perceive that the amount of matter presented for its inspection will be exceedingly small. Instead of this we think the only conditions prescribed for the admission of any given Essay to an examination and an award, are, that it should be the genuine original production of the reputed author; that it should contain important and valuable additions to our stock of knowledge concerning the subjects embraced in it; and that the name of the author should be, at the time, unknown to the committee.

The object of all prize committees should be, not so much to reward mere novelties, as to encourage all successful efforts to extend the boundaries of useful knowledge. D.

---

*Homœopathy and the University of Michigan.*

We learn from various sources that the Legislature of Michigan at its late session passed an act requiring the Board of Regents of hat State, to appoint a *Professor* of *Homœopathy* in the Medical

Department of the State University at Ann Arbor. Whether the regents will promptly obey this act of folly and thereby destroy the medical department of their University remains to be seen.

